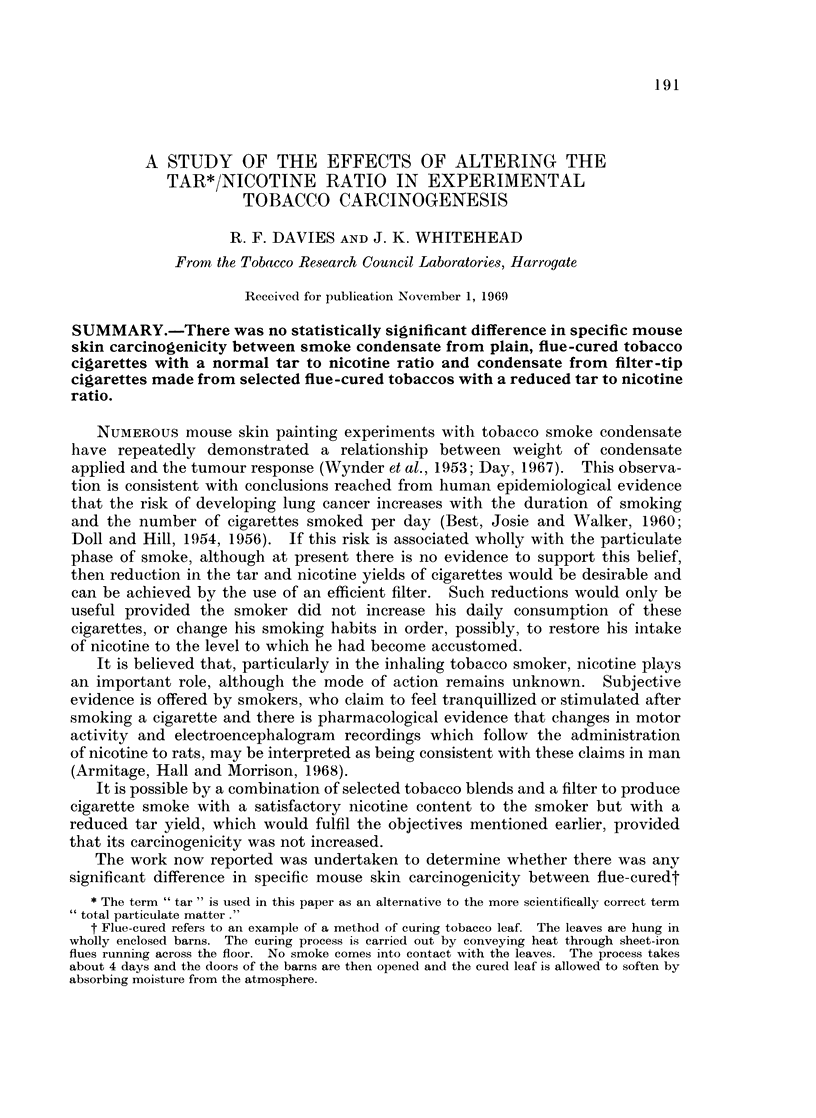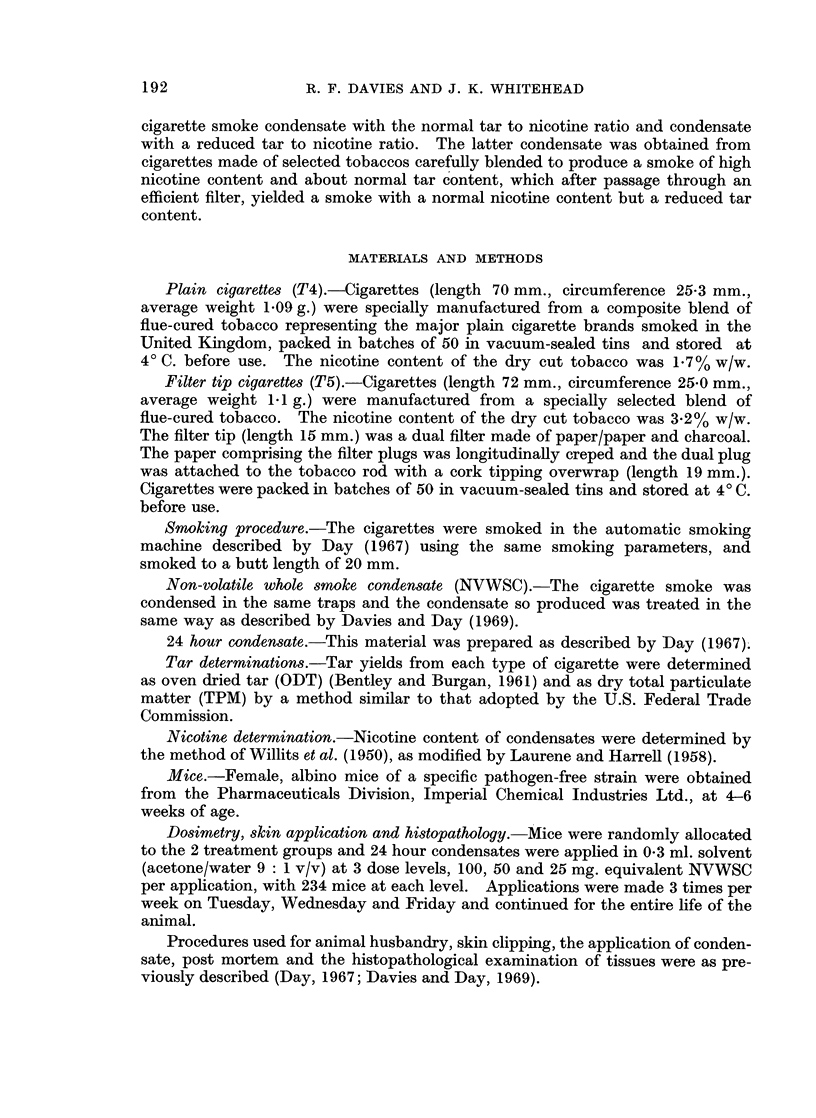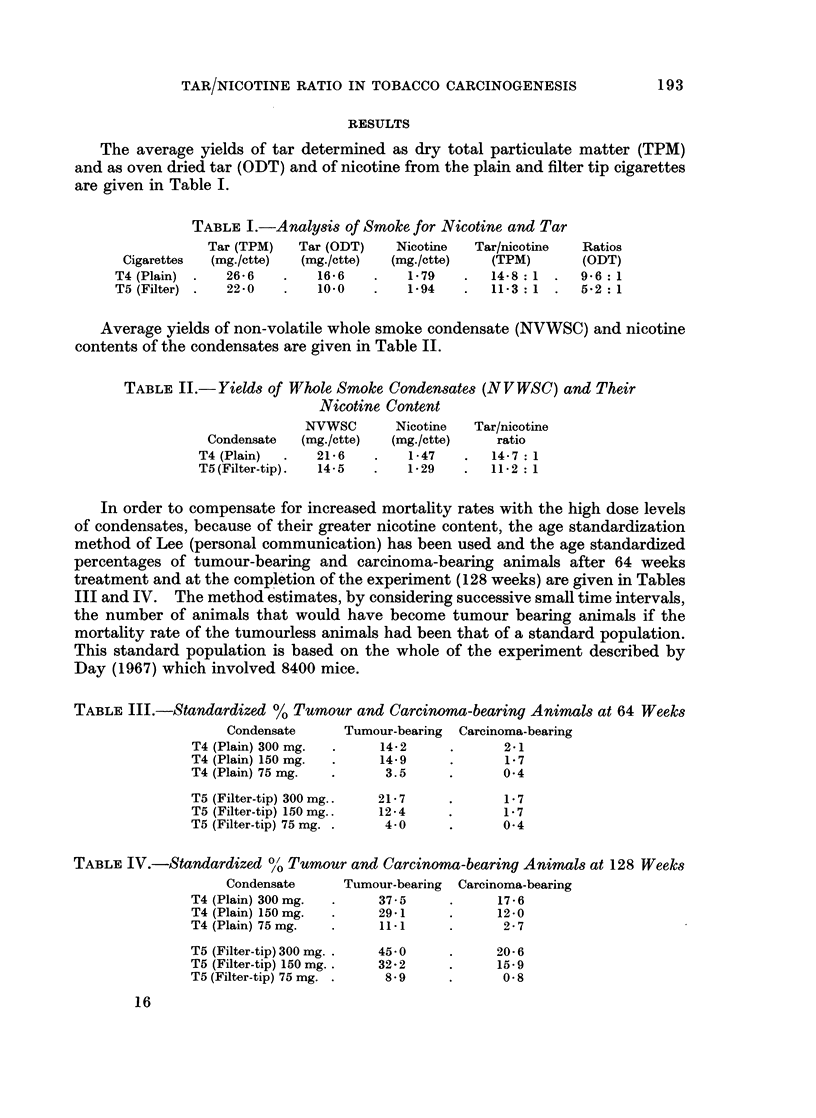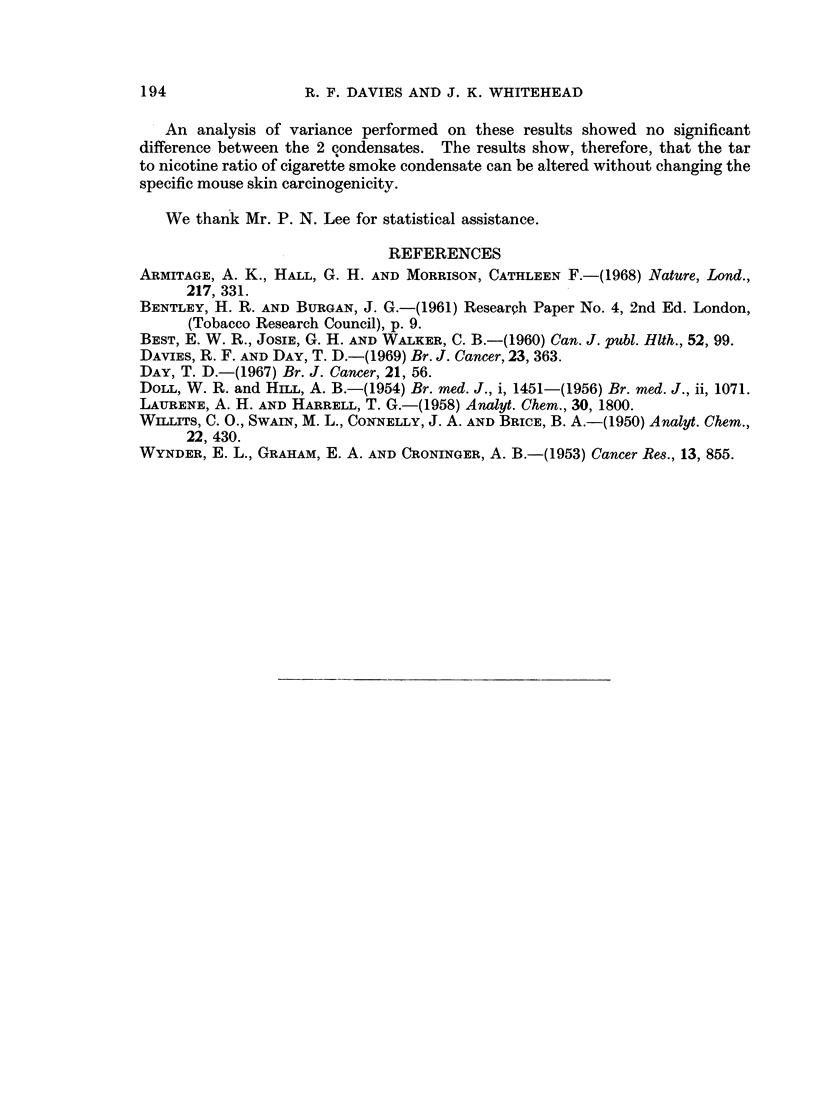# A Study of the Effects of Altering the Tar/Nicotine Ratio in Experimental Tobacco Carcinogenesis*

**DOI:** 10.1038/bjc.1970.22

**Published:** 1970-03

**Authors:** R. F. Davies, J. K. Whitehead

## Abstract

There was no statistically significant difference in specific mouse skin carcinogenicity between smoke condensate from plain, flue-cured tobacco cigarettes with a normal tar to nicotine ratio and condensate from filter-tip cigarettes made from selected flue-cured tobaccos with a reduced tar to nicotine ratio.


### A STUDY OF THE EFFECTS OF ALTERING THE TAR*/NICOTINE RATIO IN EXPERIMENTAL TOBACCO CARCINOGENESIS

R. F. DAVIES AND J. K. WHITEHEAD

*From the Tobacco Research Council Laboratories, Harrogate*

Received for publication November 1, 1969

**SUMMARY.—There was no statistically significant difference in specific mouse skin carcinogenicity between smoke condensate from plain, flue-cured tobacco cigarettes with a normal tar to nicotine ratio and condensate from filter-tip cigarettes made from selected flue-cured tobaccos with a reduced tar to nicotine ratio.**

NUMEROUS mouse skin painting experiments with tobacco smoke condensate have repeatedly demonstrated a relationship between weight of condensate applied and the tumour response (Wynder *et al.*, 1953; Day, 1967). This observation is consistent with conclusions reached from human epidemiological evidence that the risk of developing lung cancer increases with the duration of smoking and the number of cigarettes smoked per day (Best, Josie and Walker, 1960; Doll and Hill, 1954, 1956). If this risk is associated wholly with the particulate phase of smoke, although at present there is no evidence to support this belief, then reduction in the tar and nicotine yields of cigarettes would be desirable and can be achieved by the use of an efficient filter. Such reductions would only be useful provided the smoker did not increase his daily consumption of these cigarettes, or change his smoking habits in order, possibly, to restore his intake of nicotine to the level to which he had become accustomed.

It is believed that, particularly in the inhaling tobacco smoker, nicotine plays an important role, although the mode of action remains unknown. Subjective evidence is offered by smokers, who claim to feel tranquillized or stimulated after smoking a cigarette and there is pharmacological evidence that changes in motor activity and electroencephalogram recordings which follow the administration of nicotine to rats, may be interpreted as being consistent with these claims in man (Armitage, Hall and Morrison, 1968).

It is possible by a combination of selected tobacco blends and a filter to produce cigarette smoke with a satisfactory nicotine content to the smoker but with a reduced tar yield, which would fulfil the objectives mentioned earlier, provided that its carcinogenicity was not increased.

The work now reported was undertaken to determine whether there was any significant difference in specific mouse skin carcinogenicity between flue-cured†

* The term " tar " is used in this paper as an alternative to the more scientifically correct term " total particulate matter ."

† Flue-cured refers to an example of a method of curing tobacco leaf. The leaves are hung in wholly enclosed barns. The curing process is carried out by conveying heat through sheet-iron flues running across the floor. No smoke comes into contact with the leaves. The process takes about 4 days and the doors of the barns are then opened and the cured leaf is allowed to soften by absorbing moisture from the atmosphere.

cigarette smoke condensate with the normal tar to nicotine ratio and condensate with a reduced tar to nicotine ratio. The latter condensate was obtained from cigarettes made of selected tobaccos carefully blended to produce a smoke of high nicotine content and about normal tar content, which after passage through an efficient filter, yielded a smoke with a normal nicotine content but a reduced tar content.

### MATERIALS AND METHODS

*Plain cigarettes* (*T*4).—Cigarettes (length 70 mm., circumference 25·3 mm., average weight 1·09 g.) were specially manufactured from a composite blend of flue-cured tobacco representing the major plain cigarette brands smoked in the United Kingdom, packed in batches of 50 in vacuum-sealed tins and stored at 4° C. before use. The nicotine content of the dry cut tobacco was 1·7% w/w.

*Filter tip cigarettes* (*T*5).—Cigarettes (length 72 mm., circumference 25·0 mm., average weight 1·1 g.) were manufactured from a specially selected blend of flue-cured tobacco. The nicotine content of the dry cut tobacco was 3·2% w/w. The filter tip (length 15 mm.) was a dual filter made of paper/paper and charcoal. The paper comprising the filter plugs was longitudinally creped and the dual plug was attached to the tobacco rod with a cork tipping overwrap (length 19 mm.). Cigarettes were packed in batches of 50 in vacuum-sealed tins and stored at 4° C. before use.

*Smoking procedure*.—The cigarettes were smoked in the automatic smoking machine described by Day (1967) using the same smoking parameters, and smoked to a butt length of 20 mm.

*Non-volatile whole smoke condensate* (NVWSC).—The cigarette smoke was condensed in the same traps and the condensate so produced was treated in the same way as described by Davies and Day (1969).

24 *hour condensate*.—This material was prepared as described by Day (1967).

*Tar determinations*.—Tar yields from each type of cigarette were determined as oven dried tar (ODT) (Bentley and Burgan, 1961) and as dry total particulate matter (TPM) by a method similar to that adopted by the U.S. Federal Trade Commission.

*Nicotine determination*.—Nicotine content of condensates were determined by the method of Willits *et al.* (1950), as modified by Laurene and Harrell (1958).

*Mice*.—Female, albino mice of a specific pathogen-free strain were obtained from the Pharmaceuticals Division, Imperial Chemical Industries Ltd., at 4–6 weeks of age.

*Dosimetry, skin application and histopathology*.—Mice were randomly allocated to the 2 treatment groups and 24 hour condensates were applied in 0·3 ml. solvent (acetone/water 9 : 1 v/v) at 3 dose levels, 100, 50 and 25 mg. equivalent NVWSC per application, with 234 mice at each level. Applications were made 3 times per week on Tuesday, Wednesday and Friday and continued for the entire life of the animal.

Procedures used for animal husbandry, skin clipping, the application of condensate, post mortem and the histopathological examination of tissues were as previously described (Day, 1967; Davies and Day, 1969).

### RESULTS

The average yields of tar determined as dry total particulate matter (TPM) and as oven dried tar (ODT) and of nicotine from the plain and filter tip cigarettes are given in Table I.

TABLE I.—*Analysis of Smoke for Nicotine and Tar*

| Cigarettes | Tar (TPM) (mg./ctte) | Tar (ODT) (mg./ctte) | Nicotine (mg./ctte) | Tar/nicotine (TPM) | Ratios (ODT) |
|---|---|---|---|---|---|
| T4 (Plain) | 26·6 | 16·6 | 1·79 | 14·8 : 1 | 9·6 : 1 |
| T5 (Filter) | 22·0 | 10·0 | 1·94 | 11·3 : 1 | 5·2 : 1 |

Average yields of non-volatile whole smoke condensate (NVWSC) and nicotine contents of the condensates are given in Table II.

TABLE II.—*Yields of Whole Smoke Condensates (NVWSC) and Their Nicotine Content*

| Condensate | NVWSC (mg./ctte) | Nicotine (mg./ctte) | Tar/nicotine ratio |
|---|---|---|---|
| T4 (Plain) | 21·6 | 1·47 | 14·7 : 1 |
| T5 (Filter-tip) | 14·5 | 1·29 | 11·2 : 1 |

In order to compensate for increased mortality rates with the high dose levels of condensates, because of their greater nicotine content, the age standardization method of Lee (personal communication) has been used and the age standardized percentages of tumour-bearing and carcinoma-bearing animals after 64 weeks treatment and at the completion of the experiment (128 weeks) are given in Tables III and IV. The method estimates, by considering successive small time intervals, the number of animals that would have become tumour bearing animals if the mortality rate of the tumourless animals had been that of a standard population. This standard population is based on the whole of the experiment described by Day (1967) which involved 8400 mice.

TABLE III.—*Standardized % Tumour and Carcinoma-bearing Animals at* 64 *Weeks*

| Condensate | Tumour-bearing | Carcinoma-bearing |
|---|---|---|
| T4 (Plain) 300 mg. | 14·2 | 2·1 |
| T4 (Plain) 150 mg. | 14·9 | 1·7 |
| T4 (Plain) 75 mg. | 3.5 | 0·4 |
| T5 (Filter-tip) 300 mg. | 21·7 | 1·7 |
| T5 (Filter-tip) 150 mg. | 12·4 | 1·7 |
| T5 (Filter-tip) 75 mg. | 4·0 | 0·4 |

TABLE IV.—*Standardized % Tumour and Carcinoma-bearing Animals at* 128 *Weeks*

| Condensate | Tumour-bearing | Carcinoma-bearing |
|---|---|---|
| T4 (Plain) 300 mg. | 37·5 | 17·6 |
| T4 (Plain) 150 mg. | 29·1 | 12·0 |
| T4 (Plain) 75 mg. | 11·1 | 2·7 |
| T5 (Filter-tip) 300 mg. | 45·0 | 20·6 |
| T5 (Filter-tip) 150 mg. | 32·2 | 15·9 |
| T5 (Filter-tip) 75 mg. | 8·9 | 0·8 |

An analysis of variance performed on these results showed no significant difference between the 2 condensates. The results show, therefore, that the tar to nicotine ratio of cigarette smoke condensate can be altered without changing the specific mouse skin carcinogenicity.

We thank Mr. P. N. Lee for statistical assistance.

### REFERENCES

ARMITAGE, A. K., HALL, G. H. AND MORRISON, CATHLEEN F.—(1968) *Nature, Lond.*, **217**, 331.
BENTLEY, H. R. AND BURGAN, J. G.—(1961) Research Paper No. 4, 2nd Ed. London, (Tobacco Research Council), p. 9.
BEST, E. W. R., JOSIE, G. H. AND WALKER, C. B.—(1960) *Can. J. publ. Hlth.*, **52**, 99.
DAVIES, R. F. AND DAY, T. D.—(1969) *Br. J. Cancer*, **23**, 363.
DAY, T. D.—(1967) *Br. J. Cancer*, **21**, 56.
DOLL, W. R. and HILL, A. B.—(1954) *Br. med. J.*, i, 1451—(1956) *Br. med. J.*, ii, 1071.
LAURENE, A. H. AND HARRELL, T. G.—(1958) *Analyt. Chem.*, **30**, 1800.
WILLITS, C. O., SWAIN, M. L., CONNELLY, J. A. AND BRICE, B. A.—(1950) *Analyt. Chem.*, **22**, 430.
WYNDER, E. L., GRAHAM, E. A. AND CRONINGER, A. B.—(1953) *Cancer Res.*, **13**, 855.